# Sulphamethoxazole-trimethoprim induced fixed drug eruption in a patient with Chronic Granulomatous Disease

**DOI:** 10.5339/qmj.2022.fqac.6

**Published:** 2022-04-07

**Authors:** Sherin Rahim Thalappil, Maryam A Al-Nesf

**Affiliations:** ^1^Adult Allergy and Immunology Section, Department of Medicine, Hamad Medical Corporation, Doha, Qatar E-mail: SThalappil@hamad.qa

## Abstract

Chronic granulomatous disease (CGD) is a known Primary immunodeficiency disease that results in recurrent, life-threatening bacterial, fungal infections and granuloma formation which requires lifelong antibacterial and antifungal prophylaxis. Sulphamethoxazole-trimethoprim (TMP-SMX/Septrin) is the prophylactic antibacterial drug of choice.^1^ Adverse drug reactions, including Fixed Drug Eruption (FDE) to TMP-SMX in CGD patients, are challenging as it may result in serious complications and difficulties in management.

A seventeen-year-old Qatari female diagnosed with CGD was maintained on prophylactic TMP-SMX and itraconazole since childhood. She developed bullous skin lesions involving the face, neck, and trunk sparing the limbs. The skin lesion was confirmed to be FDE by skin biopsy. TMP-SMX was discontinued as it was suspected to be the causative agent. Atovaquone 1500 mg and clarithromycin 250 mg daily were started as an alternative, but the patient could not tolerate them. TMP-SMX slow graded challenge and desensitization were performed; however, the patient developed similar lesions in the previous affected areas on day 3 of desensitization, which responded well to discontinuation of TMP-SMX along with administration of steroid. FDE to TMP-SMX as the causative agent was confirmed. An MDT meeting was conducted to evaluate other available options for treatment. Also, the case was discussed with international immunology colleagues. The recommendation was to go for bone marrow transplantation to treat the primary disease. Six years post transplants, the patient is doing well and is not on any medications apart from the hormonal replacement therapy patch.

CGD predisposes to several gram-positive, gram-negative bacterial (Staphylococcus aureus, Burkholderia cepacia complex, Serratia marcescens, Nocardia species) and fungal infections.^2^ TMP-SMX is an ideal antibiotic as it is relatively cheap with good coverage and orally available. In the medical literature, desensitization can be tried in FDE, especially if there is no alternative for the needed drug. FDE signs should be observed when administered TMP-SMX.
